# Social capital and regional influences: key predictors of unmet dental care needs among older adults in Korea

**DOI:** 10.4178/epih.e2025025

**Published:** 2025-05-07

**Authors:** Ji-Yeon Lim, Ju-Mi Lee, Hae-Sung Nam

**Affiliations:** 1 Department of Public Health, Graduate School, Chungnam National University, Daejeon, Korea; 2 Department of Preventive Medicine, Chungnam National University College of Medicine, Daejeon, Korea

**Keywords:** Dental care for aged, Dental health services, Social capital, Health services accessibility, Oral health

## Abstract

**OBJECTIVES:**

Access to dental services is essential for improving quality of life, and social capital plays a key role in facilitating that access. This study aimed to identify individual-level and regional-level factors, including social capital, that predict unmet dental care needs among older adults.

**METHODS:**

We analyzed data from 59,414 older adults obtained from the 2023 Korea Community Health Survey and the Korean Statistical Information Service, employing a 2-level multilevel model. The dependent variables comprised 3 types of unmet dental care needs: overall, due to lack of acceptability, and due to economic reasons. Twelve independent variables, including social capital and other individual and regional factors, were examined.

**RESULTS:**

The prevalence of unmet needs was 14.15% overall, 8.70% for acceptability reasons, and 4.85% for economic reasons. Lower individual social capital was associated with higher odds of unmet dental care needs, whereas regional social capital factors demonstrated no significant association. Residing in regions with higher fiscal independence ratios was related to an increased likelihood of economic unmet needs (odds ratio [OR], 1.29; 95% confidence interval [CI], 1.09 to 1.52). In contrast, a greater density of dentists per 10,000 population was inversely associated with overall and acceptability-related unmet needs (OR, 0.82 for both; 95% CI, 0.73 to 0.92 and 0.73 to 0.93, respectively).

**CONCLUSIONS:**

Individual social capital and specific regional factors—namely, fiscal independence and density of dentists—may represent important determinants of unmet dental care needs among older adults. Policy interventions aimed at reducing unmet needs should consider these variables.

## GRAPHICAL ABSTRACT

[Fig f2-epih-47-e2025025]

## Key Message

• Lower social capital (lower levels of trust in neighbors, less frequent contact, absence of a spouse, and lack of social participation) increases the risk of unmet dental care needs among older adults.

• Higher regional fiscal independence increases unmet dental care needs due to economic reasons, while a greater density of dentists reduces overall unmet dental care needs and those due to lack of acceptability.

## INTRODUCTION

Oral health, including the number of remaining teeth and functional oral status, is significantly associated with disability and mortality and has been linked to delayed cognitive decline in older adults [1]. In a 3-year longitudinal study of 2,011 individuals aged 65 years and older, those with oral frailty exhibited a 2.09-fold higher hazard ratio for mortality compared to those without such frailty [2]. Access to dental services—a key means of preserving oral health—is crucial for reducing the prevalence of oral diseases and their associated health burdens, thus improving overall quality of life [3,4].

Unmet dental care needs indicate inequity in accessing dental care services [4,5]. They occur when individuals require dental treatment but cannot obtain it due to economic, physical, or geographical barriers [6]. These unmet needs reflect underlying socioeconomic disparities, and people with lower incomes and education levels report them particularly often [5]. Among the 26 Organization for Economic Cooperation and Development (OECD) countries, the average rate of unmet dental care needs was 3.4%, with Portugal having the highest rate at 9.0% [7]. However, in Korea, the rate of unmet dental care needs has reached 33.1% [8], approximately 10 times the OECD average. Therefore, the phenomenon of high unmet dental care needs in Korea merits careful attention—particularly as it exists in spite of the national health insurance system and policies providing coverage for dentures and implants to older adults.

Social capital is defined by the OECD as a network of shared norms, values, and understandings that promote cooperation within and between groups [9]. According to the Legatum Prosperity Index, Korea’s social capital score—which measures trust in others and in government—was 51.59%, ranking 107th out of 167 countries, behind Denmark (82.56%), Sweden (78.29%), and Finland (77.42%) [10]. Social participation and interpersonal networks predict both mental and physical health [11]. Specifically, socially isolated older adults had, on average, 1.4 times fewer remaining teeth and experienced poorer oral and general health [12]. These findings underscore the importance of social capital in shaping oral health status and dental care utilization, particularly in an aging society characterized by increasingly nuclear family structures.

However, existing studies on unmet dental care needs among older adults have primarily focused on individual-level variables [13-17]. Although some research has examined the relationship between unmet dental care needs and social capital, this too has been limited to the individual level [16]. Other studies have explored the relationship between social capital and dental utilization [18,19], but dental utilization—the actual use of dental services—differs fundamentally from unmet dental care needs, which occur when individuals perceive an inability to access necessary treatment. Thus, utilization and unmet needs represent opposite aspects of access. Furthermore, additional research is required to understand the underlying causes of high rates of unmet dental care needs, particularly in countries like Korea. A prior Korean study [20] investigated reasons for unmet dental care needs but did not adequately address how social capital influences these patterns. Therefore, the present study aimed to perform a multilevel analysis using the Korea Community Health Survey (KCHS), conducted at the district level, and the Korean Statistical Information Service (KOSIS) to identify individual and regional characteristics associated with unmet dental care needs for specific reasons. Additionally, this study examines the potential association of factors such as social capital with unmet dental care needs, which have not been addressed in previous research [16,20].

## MATERIALS AND METHODS

### The Korea Community Health Survey and study sampling

This study employed multilevel cross-sectional analyses using data from the KCHS and the KOSIS. The KCHS, a health survey conducted annually by the Korea Disease Control and Prevention Agency since 2008 [21], surveys adults aged 19 years and older across all municipalities in Korea. To ensure a representative sample, an average of 900 people per public health center were selected via multistage probability sampling. Trained regional interviewers collected data through one-on-one electronic interviews. As of 2023, the survey included 231,752 participants. In the present research, after excluding individuals under 65 years of age, participants with missing responses to questions about unmet dental care needs, and those with incomplete values for other variables, the final study population comprised 59,414 older adults.

### Dependent variables

We classified 3 types of unmet dental care needs as dependent variables: overall needs, those due to lack of acceptability, and those due to economic reasons. Self-reported unmet needs were measured using the KCHS question: “In the last year, have you been unable to go to the dental clinic or the hospital when you needed to?” A “yes” response indicated overall unmet needs. Among respondents reporting unmet needs, we identified the most frequently cited reasons and categorized them into lack of acceptability and economic reasons [14,22]. Acceptability-related needs included barriers stemming from personal circumstances or perceptions—such as “no time,” “mildness of symptoms,” or “fear of treatment”—whereas economic unmet needs were those attributed to financial constraints, including the response of “economic reasons” [20].

### Independent variables (individual level)

Individual-level variables were divided into 3 categories: socioeconomic status, oral health, and social capital. Socioeconomic variables comprised gender (men, women), education level (middle school or lower vs. high school or higher), and household income. Oral health variables included subjective oral health status (good/bad) and chewing difficulty (yes/no). Social capital status, a focus of this study, was assessed in cognitive and structural dimensions [23]. Cognitive social capital—reflecting shared norms, values, perceptions, attitudes, and beliefs—is measured by items such as “Do you think most people can be trusted?” [24]. Accordingly, we extracted neighborhood trust (yes/no) and frequency of contact (high/low) from the KCHS. Structural social capital captures observable aspects of social organization, including network density and civic engagement [24], with marital status indicating a key form of close structural support [25]. Therefore, we included social participation (yes or no) and marital status (having a spouse: yes/no) from the KCHS data.

### Independent variables (regional level)

Regional-level variables were grouped into 3 conceptual domains—the economic environment, dental care resources, and regional social capital—to reflect the pathways through which they shape individual health environments, based on data from KOSIS. We used the fiscal independence ratio (high vs. low) as our economic indicator and dentist density (number of dentists per 10,000 population; high vs. low) to represent dental care resources [14,22]. Regional social capital was captured by volunteer engagement (number of volunteers per 1,000 population; high vs. low) and civic infrastructure (number of non-profit organizations per 100,000 population; high vs. low) [26,27].

### Statistical analysis

**Figure 1.**
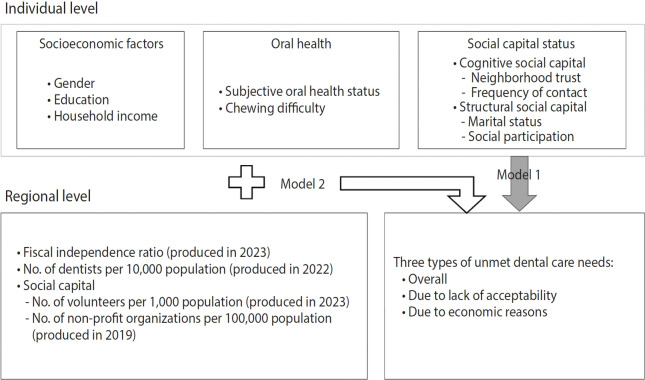
Conceptual model of individual- and regional-level characteristics related to unmet dental care needs. The gray arrows represent model 1, which includes individual factors, while the white arrows represent model 2, which incorporates individual and regional factors.

Because the KCHS employed a complex sampling design, all analyses incorporated the associated sample weights [21]. Multilevel logistic regression analysis was applied to 59,414 individuals (level 1) nested within 229 districts (level 2) to examine associations between regional factors and unmet dental care needs, adjusting for individual-level characteristics. Three models were specified: the null model; model 1, which included individual factors only; and model 2, which incorporated the variables in model 1 as well as regional factors (Figure 1). Model evaluation proceeded in 3 steps. First, we assessed multicollinearity, confirming that all variance inflation factors remained below 15. Second, to assess the need for multilevel modeling, we calculated the intraclass correlation coefficient (ICC), computed as σ^2^_μ_/(σ^2^_μ_+3.29) [28,29]. The ICC indicates the proportion of total variance in unmet dental care needs attributable to district-level differences (level 2). Third, we compared model fit using the -2 log-likelihood ratio (deviance), with lower values indicating a more suitable result. Complex‐sample frequency distributions were generated via PROC SURVEYFREQ and multilevel analyses were performed using PROC GLIMMIX, both in SAS version 9.4 (SAS Institute Inc., Cary, NC, USA).

### Ethics statement

The Institutional Review Board of Chungnam National University exempted this study from ethical review (IRB No. 202410-SB-137-01), as it was conducted using secondary data without identifiable personal information.

## RESULTS

### General characteristics of the study population

Table 1 presents the prevalence of unmet dental care needs by individual-level and regional-level characteristics of the complex survey sample (n=59,414), as assessed by χ^2^ tests. The prevalence of overall unmet needs was 14.15%, the rate of unmet needs due to lack of acceptability was 8.70%, and that of unmet needs due to economic reasons was 4.85%.

At the individual level, several characteristics were significantly associated with unmet dental care needs. Women, individuals with lower educational attainment, and those with lower household incomes were more likely to report each of the 3 types of unmet needs (p<0.001). Similarly, participants with poor subjective oral health and those experiencing chewing difficulties exhibited a higher prevalence across the 3 categories (p<0.001 for all types). Regarding social capital, older adults without a spouse, those exhibiting low interpersonal trust, those with infrequent social contacts, and those not participating in social groups also showed significantly elevated rates of unmet dental care needs (p<0.001 for all types).

At the regional level, living in an area with fewer dentists per 10,000 population was associated with a significantly higher prevalence of overall, acceptability-related, and economic unmet needs (p<0.001, <0.01, and <0.05, respectively). Residents of communities with fewer volunteers per 1,000 population experienced higher economic unmet needs (p<0.05), but no significant differences were observed for overall or acceptability-related unmet needs. Similarly, individuals in areas with fewer non-profit organizations per 100,000 population reported higher rates across all 3 unmet-need categories (p<0.05 for overall and acceptability-related unmet needs; p<0.01 for economic unmet needs).

### Factors related to the 3 types of unmet dental care needs

Table 2 presents the multilevel logistic regression results for unmet dental needs overall, due to a lack of acceptability, and due to economic reasons. In the null model for overall unmet needs, the ICC was 0.04, indicating that 4.0% of the total variance in unmet dental care needs was attributable to regional differences. At the individual level, all variables except education level were significantly associated with overall unmet needs. Poor subjective oral health (odds ratio [OR], 2.14; 95% confidence interval [CI], 1.94 to 2.35) and chewing difficulty (OR, 2.96; 95% CI, 2.82 to 3.12) were both strongly associated with overall unmet needs. Regarding social capital factors, the absence of a spouse, low neighborhood trust, infrequent social contact, and non-participation in social groups all increased the odds of overall unmet needs. Regarding regional social capital indicators, volunteer density per 1,000 population and nonprofit organization density per 100,000 population were not significantly associated with overall unmet needs. However, higher density of dentists (dentists per 10,000 population) was inversely associated (OR, 0.82 for high vs. low density; 95% CI, 0.73 to 0.92).

For acceptability-related unmet needs, the ICC of the null model was 0.04, with a variance of 0.15. At the individual level, most socioeconomic, oral health, and social capital variables were significantly associated with acceptability-related unmet needs, with the exceptions of household income, marital status, and social participation. Regarding individual social capital, lack of neighborhood trust (OR, 1.17; 95% CI, 1.08 to 1.26) and low contact frequency (OR, 1.12; 95% CI, 1.05 to 1.20) were associated with increased odds of unmet needs due to acceptability. No regional social capital indicators demonstrated significance. In the fully adjusted model—including individual-level covariates—higher dentist density per 10,000 population was inversely associated with acceptability-related unmet needs (OR, 0.82; 95% CI, 0.73 to 0.93).

For economic unmet needs, the ICC of the null model was 0.06. At the individual level, all variables except gender were significantly associated with economic unmet needs. Compared with those in the highest income bracket (≥3,000,000 Korean won [KRW]), participants in the lowest bracket (<1,000,000 KRW) had markedly higher odds of economic unmet needs (OR, 2.86; 95% CI, 2.46 to 3.32). Regarding oral health indicators, both subjective oral health status (OR, 3.37; 95% CI, 2.59 to 4.40) and chewing difficulty (OR, 5.16; 95% CI, 4.66 to 5.71) were strong predictors of economic unmet needs. Individual social capital factors—namely low neighborhood trust, infrequent social contact, absence of a spouse, and non-participation in social groups—also increased the likelihood of experiencing unmet needs for economic reasons. No regional social capital indicators demonstrated significance. However, residing in a region with a higher fiscal independence ratio was associated with greater odds of economic unmet needs (OR, 1.29; 95% CI, 1.09 to 1.52).

## DISCUSSION

This multilevel analysis identified both individual‐level and regional‐level determinants of unmet dental care needs among older adults across 3 categories: overall, due to lack of acceptability, and due to economic reasons. At the individual level, lower neighborhood trust and infrequent social contact—key indicators of diminished social capital—were associated with higher odds of experiencing unmet needs in all 3 categories. Regionally, a higher fiscal independence ratio was linked to greater economic unmet needs, while a greater density of dentists per 10,000 population was associated with reduced overall and acceptability‐related unmet needs.

The main findings of this study indicate that individual social capital factors—including lower interpersonal trust and infrequent social interactions—were significantly associated with higher odds of all 3 categories of unmet dental care needs. This observation, which we believe to be novel, suggests that increasing social capital may aid in reducing these unmet needs. While no study has directly modeled the pathway by which high social capital reduces unmet dental care needs, related research suggests plausible mechanisms [12,24,30-34]. For example, greater trust in others has been linked to reduced dental anxiety and increased dental service utilization [30,31], and more social interaction has been associated with a higher likelihood of dental care use [32]. These findings imply that social belonging and perceived safety confer psychological stability [12,24,33], and stronger community trust fosters a sense of purpose and identity in older adults, positively influencing health outcomes [34]. Consequently, practical support, emotional stability, and access to health-related information obtained through social capital may lower barriers to dental care and ultimately decrease unmet dental care needs. In contrast, regional social capital metrics did not show significant associations, consistent with prior findings [19,26,35]. In Korea specifically, high residential mobility may impede the formation of stable, region-based social capital conducive to dental care access [36].

Furthermore, among potential regional determinants of unmet dental care needs, dentist density per 10,000 population was inversely associated with both overall and acceptability-related unmet needs, while a higher fiscal independence ratio corresponded to an increased likelihood of economic unmet needs.

This inverse relationship with dentist density likely reflects that regions with more dental professionals can offer shorter waiting times for appointments, thereby alleviating acceptability-related barriers to care [37,38].

Regarding the regional economic environment and unmet dental care needs, previous research reported that a 1% increase in regional poverty was associated with a 5% rise in unmet needs [26]. In contrast, our finding that higher fiscal independence ratios correlate with greater economic unmet needs diverges from these earlier results [26]. This discrepancy may reflect the distinct healthcare context in Korea. In 2021, dental expenditures accounted for 5.7% of total healthcare spending, compared with 1.6% in Finland, 3.5% in the United Kingdom, and 4.0% in the United States [39]. Moreover, insurance coverage for dental services in Korea remains relatively low (approximately 30%) versus medical services (about 50%) [39]. Regions with higher fiscal independence often possess more dental care resources [40], which can intensify market competition and prompt providers to promote uninsured services [41]. Such practices may amplify unmet dental care needs resulting from economic burdens, particularly for lower-income residents.

This study has several limitations. First, because it relied on cross-sectional data, it could not establish causal relationships between unmet dental care needs and their influencing factors. Therefore, longitudinal research, such as cohort studies, is needed to clarify temporal sequences and causality. Second, we did not include every factor potentially associated with unmet dental care needs, so future research should incorporate additional relevant variables. Third, while the survey-based indicator of unmet dental care needs is a simple measure that can represent subjective healthcare utilization and health equity, it lacks clinical detail and may differ from objective dental diagnosis. Revising survey items to include specific physical and psychological issues and developing standardized assessment tools would help reduce this gap. Finally, district-level indicators of regional social capital in Korea are currently limited; more comprehensive measures at this level should be designed, and ongoing research should explore how regional social capital influences unmet dental care needs.

In conclusion, our results suggest that both individual social capital and regional environmental factors—specifically the fiscal independence ratio and dentist density—are key to reducing unmet dental care needs among community-dwelling older adults. Policy interventions designed to address these needs should therefore consider both of these sets of variables.

## Figures and Tables

**Figure f2-epih-47-e2025025:**
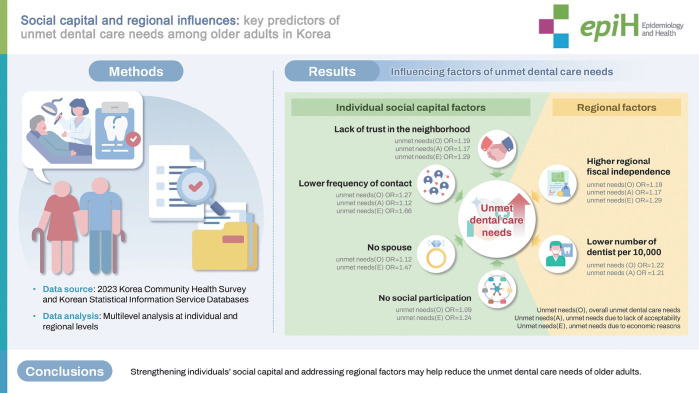


**Table 1.** Distribution of unmet dental care needs by general characteristics among older adults in Korea

Characteristics	Unmet needs (O)		Unmet needs (A)		Unmet needs (E)	
	Unmet	Met	Unmet	Met	Unmet	Met
Individual level (1-level)						
Socioeconomic status						
Gender						
Men	2,955 (11.69)	21,827 (88.31)	1,724 (7.21)	21,827 (92.80)	882 (4.28)	21,827 (95.72)
Women	5,928 (16.12)	28,704 (83.88)	3,202 (9.93)	28,704 (90.07)	1,632 (5.33)	28,704 (94.67)
p-value	<0.001		<0.001		<0.001	
Education						
High school or higher	2,072 (11.09)	16,377 (88.91)	1,399 (7.83)	16,377 (92.17)	463 (2.90)	16,377 (97.10)
Middle school or lower	6,811 (16.19)	34,154 (83.81)	3,527 (9.31)	34,154 (90.69)	2,051 (6.19)	34,154 (93.81)
p-value	<0.001		<0.001		<0.001	
Household income (10^3^ KRW)						
≥3,000	1,652 (10.59)	13,671 (89.41)	1,219 (8.19)	13,671 (91.81)	262 (1.96)	13,671 (98.04)
1,000-3,000	3,606 (13.47)	23,149 (86.53)	2,158 (8.36)	23,149 (91.64)	968 (4.73)	23,149 (95.27)
≤1,000	3,625 (21.33)	13,711 (78.67)	1,549 (10.40)	13,711 (89.60)	1,284 (10.00)	13,711 (90.00)
p-value	<0.001		<0.001		<0.001	
Oral health status						
Subjective oral health status						
Good	507 (5.10)	9,216 (94.90)	379 (3.95)	9,216 (96.05)	60 (0.68)	9,216 (99.32)
Bad	8,376 (16.06)	41,315 (83.94)	4,547 (9.77)	41,315 (90.23)	2,454 (5.80)	41,315 (94.20)
p-value	<0.001		<0.001		<0.001	
Chewing difficulty						
No	3,324 (8.66)	35,041 (91.34)	2,287 (6.24)	35,041 (93.76)	573 (1.86)	35,041 (98.14)
Yes	5,559 (25.97)	15,490 (74.03)	2,639 (14.65)	15,490 (85.35)	1,941 (11.98)	15,490 (88.02)
p-value	<0.001		<0.001		<0.001	
Social capital status						
Neighborhood trust						
Yes	6,715 (13.15)	40,322 (86.85)	3,785 (8.21)	40,322 (91.79)	1,767 (4.19)	40,322 (95.81)
No	2,168 (16.83)	10,209 (83.17)	1,141 (10.06)	10,209 (89.94)	747 (6.68)	10,209 (93.32)
p-value	<0.001		<0.001		<0.001	
Frequency of contact						
High	4,452 (12.43)	28,617 (87.57)	2,655 (8.09)	28,617 (91.91)	1,006 (3.45)	28,617 (96.55)
Low	4,431 (15.57)	21,914 (84.43)	2,271 (9.22)	21,914 (90.78)	1,508 (6.03)	21,914 (93.98)
p-value	<0.001		<0.001		<0.001	
Marital status						
Yes	4,807 (12.11)	33,534 (87.89)	3,061 (8.24)	33,534 (91.76)	1,122 (3.42)	33,534 (96.58)
No	4,076 (18.38)	16,997 (81.62)	1,865 (9.72)	16,997 (90.28)	1,392 (7.91)	16,997 (92.09)
p-value	<0.001		<0.001		<0.001	
Social participation						
Yes	5,111 (12.28)	33,867 (87.72)	3,158 (8.14)	33,867 (91.86)	1,244 (3.64)	33,867 (96.36)
No	3,772 (18.30)	16,664 (81.70)	1,768 (10.02)	16,664 (89.98)	1,270 (7.63)	16,664 (92.37)
p-value	<0.001		<0.001		<0.001	
Regional level (2-level)						
Fiscal independence ratio						
Low	5,642 (14.57)	30,453 (85.43)	3,052 (8.68)	30,453 (91.32)	1,490 (4.79)	30,453 (95.21)
High	3,241 (13.91)	20,078 (86.09)	1,874 (8.71)	20,078 (91.29)	1,024 (4.88)	20,078 (95.12)
p-value	0.064		0.920		0.678	
No. of dentists per 10,000 population						
Low	5,217 (15.58)	27,143 (84.42)	2,848 (9.31)	27,143 (90.69)	1,397 (5.17)	27,143 (94.83)
High	3,666 (13.45)	23,388 (86.55)	2,078 (8.41)	23,388 (91.59)	1,117 (4.70)	23,388 (95.30)
p-value	<0.001		<0.01		<0.05	
No. of volunteers per 1,000 population						
Low	4,076 (14.30)	23,306 (85.70)	2,259 (8.85)	23,306 (91.15)	1,229 (5.04)	23,306 (94.96)
High	4,807 (13.90)	27,225 (86.10)	2,667 (8.46)	27,225 (91.54)	1,285 (4.55)	27,225 (95.45)
p-value	0.271		0.193		<0.05	
No. of non-profit organizations per 100,000 population						
Low	4,034 (14.51)	22,925 (85.49)	2,288 (8.99)	22,925 (91.01)	1,199 (5.13)	22,925 (94.87)
High	4,849 (13.62)	27,606 (86.38)	2,638 (8.28)	27,606 (91.72)	1,315 (4.45)	27,606 (95.55)
p-value	<0.05		<0.05		<0.01	
Total	8,883 (14.15)	50,531 (85.85)	4,926 (8.70)	50,531 (91.30)	2,514 (4.85)	50,531 (95.15)

Values are presented as number (weighted %). Unmet needs (O), overall unmet dental care needs; Unmet needs (A), unmet needs due to lack of acceptability; Unmet needs (E), unmet needs due to economic reasons; KRW, Korean won.

**Table 2.** Results of multilevel analysis of unmet dental care needs in older Korean adults

Variables	Unmet needs (O)		Unmet needs (A)		Unmet needs (E)	
	Model 1	Model 2	Model 1	Model 2	Model 1	Model 2
Individual level (1-level)						
Socioeconomic status						
Gender						
Men	Reference	Reference	Reference	Reference	Reference	Reference
Women	1.37 (1.30, 1.45)^***^	1.37 (1.30, 1.45)^***^	1.45 (1.35, 1.55)^***^	1.45 (1.35, 1.55)^***^	1.06 (0.96, 1.17)	1.06 (0.96, 1.17)
Education						
High school or higher	Reference	Reference	Reference	Reference	Reference	Reference
Middle school or lower	0.98 (0.92, 1.04)	0.98 (0.92, 1.04)	0.91 (0.84, 0.98)^*^	0.91 (0.84, 0.98)^*^	1.16 (1.03, 1.31)^*^	1.18 (1.05, 1.33)^**^
Household income (10^3^ KRW)						
≥3,000	Reference	Reference	Reference	Reference	Reference	Reference
1,000-3,000	1.13 (1.05, 1.20)^***^	1.12 (1.05, 1.20)^***^	0.95 (0.88, 1.03)	0.95 (0.88, 1.03)	1.89 (1.64, 2.18)^***^	1.91 (1.66, 2.21)^***^
≤1,000	1.39 (1.29, 1.50)^***^	1.39 (1.29, 1.50)^***^	0.96 (0.87, 1.05)	0.95 (0.87, 1.04)	2.81 (2.42, 3.26)^***^	2.86 (2.46, 3.32)^***^
Oral health status						
Subjective oral health status						
Good	Reference	Reference	Reference	Reference	Reference	Reference
Bad	2.14 (1.94, 2.36)^***^	2.14 (1.94, 2.35)^***^	1.95 (1.74, 2.18)^***^	1.95 (1.74, 2.18)^***^	3.37 (2.59, 4.40)^***^	3.37 (2.59, 4.40)^***^
Chewing difficulty						
No	Reference	Reference	Reference	Reference	Reference	Reference
Yes	2.96 (2.82, 3.12)^***^	2.96 (2.82, 3.12)^***^	2.31 (2.17, 2.47)^***^	2.31 (2.17, 2.47)^***^	5.13 (4.64, 5.68)^***^	5.16 (4.66, 5.71)^***^
Social capital status						
Neighborhood trust						
Yes	Reference	Reference	Reference	Reference	Reference	Reference
No	1.19 (1.13, 1.27)^***^	1.19 (1.13, 1.27)^***^	1.17 (1.08, 1.26)^***^	1.17 (1.09, 1.26)^***^	1.31 (1.18, 1.45)^***^	1.29 (1.17, 1.43)^***^
Frequency of contact						
High	Reference	Reference	Reference	Reference	Reference	Reference
Low	1.26 (1.20, 1.33)^***^	1.27 (1.20, 1.33)^***^	1.12 (1.05, 1.19)^***^	1.12 (1.05, 1.20)^***^	1.68 (1.53, 1.84)^***^	1.66 (1.51, 1.82)^***^
Marital status						
Yes	Reference	Reference	Reference	Reference	Reference	Reference
No	1.12 (1.06, 1.19)^***^	1.12 (1.06, 1.19)^***^	0.94 (0.87, 1.01)	0.94 (0.88, 1.01)	1.47 (1.34, 1.63)^***^	1.47 (1.33, 1.62)^***^
Social participation						
Yes	Reference	Reference	Reference	Reference	Reference	Reference
No	1.09 (1.04, 1.15)^***^	1.09 (1.04, 1.15)^***^	0.95 (0.89, 1.02)	0.95 (0.89, 1.02)	1.23 (1.13, 1.35)^***^	1.24 (1.13, 1.35)^***^
Regional level (2-level)						
Fiscal independence ratio						
Low	Reference	Reference	Reference	Reference	Reference	Reference
High	Reference	1.04 (0.92, 1.17)	Reference	1.03 (0.90, 1.17)	Reference	1.29 (1.09, 1.52)^**^
No. of dentists per 10,000 population						
Low	Reference	Reference	Reference	Reference	Reference	Reference
High	Reference	0.82 (0.73, 0.92)^***^	Reference	0.82 (0.73, 0.93)^**^	Reference	0.92 (0.79, 1.08)
No. of volunteers per 1,000 population						
Low	Reference	Reference	Reference	Reference	Reference	Reference
High	Reference	0.94 (0.83, 1.05)	Reference	0.97 (0.86, 1.10)	Reference	0.88 (0.75, 1.03)
No. of non-profit organizations per 100,000 population						
Low	Reference	Reference	Reference	Reference	Reference	Reference
High	Reference	0.98 (0.87, 1.10)	Reference	0.96 (0.85, 1.09)	Reference	0.94 (0.80, 1.11)
ICC^1^	0.04	0.04	0.04	0.04	0.07	0.06
-2 log likelihood	45,593.14	45,580.68	31,696.54	31,686.49	16,980.88	16,964.06

Values are presented as odds ratio (95% confidence interval). Unmet needs (O), overall unmet dental care needs; Unmet needs (A), unmet needs due to lack of acceptability; Unmet needs (E), unmet needs due to economic reasons; KRW, Korean won; ICC, intraclass correlation coefficient.

1 In the null model, the ICC was 0.04 for unmet needs (O), 0.04 for (A), and 0.06 for (E).

* p<0.05,

** p<0.01,

*** p<0.001.
